# Qi-Shen-Yi-Qi Dripping Pills for the Secondary Prevention of Myocardial Infarction: A Randomised Clinical Trial

**DOI:** 10.1155/2013/738391

**Published:** 2013-07-14

**Authors:** Hongcai Shang, Junhua Zhang, Chen Yao, Baoyan Liu, Xiumei Gao, Ming Ren, Hongbao Cao, Guohua Dai, Weiliang Weng, Sainan Zhu, Hui Wang, Hongjuan Xu, Boli Zhang

**Affiliations:** ^1^Tianjin University of Traditional Chinese Medicine, 312 Anshanxi Road, Nankai District, Tianjin 300193, China; ^2^Peking University First Hospital, Beijing 100034, China; ^3^Chinese Academy of Traditional Chinese Medicine, Beijing 100700, China; ^4^Affiliated Hospital of Shandong University of Traditional Chinese Medicine, Jinan, Shandong 250011, China; ^5^Xiyuan Hospital, Beijing 100091, China

## Abstract

*Background.* Several types of drugs have been recommended for the secondary prevention of myocardial infarction (MI). However, these conventional strategies have several limitations, such as low adherence, high cost, and side effects during long time use. Novel approaches to this problem are still needed. This trial aimed to test the effectiveness and safety of Qi-Shen-Yi-Qi Dripping Pills (QSYQ), a multi-ingredient Chinese patent medicine, for the secondary prevention of MI. *Methods and Findings*. A total of 3505 eligible patients were randomly assigned to QSYQ group (1746 patients) or aspirin group (1759). Patients took their treatments for 12 months. The final follow-up visit took place 6 months after the end of the trial drugs. The 12-month and 18-month estimated incidences of the primary outcome were 2.98% and 3.67%, respectively, in the QSYQ group. The figures were 2.96% and 3.81% in the aspirin group. No significant difference was identified between the groups. *Conclusions*. This trial did not show significant difference of primary and secondary outcomes between aspirin and QSYQ in patients who have had an MI. Though inconclusive, the result suggests that QSYQ has similar effects to aspirin in the secondary prevention of MI.

## 1. Introduction

Acute myocardial infarction is a leading cause of death worldwide [1]. More than 7 million people a year have a myocardial infarction (MI) [2]. Over the past three decades, MI has emerged from an illness seen predominantly in developed countries to more common in developing countries [2–4]. Progresses in emergency management have led to substantial reductions in the mortality rate of acute MI [2]. However, survivors from acute MI remain at greatly increased risk of serious vascular events [2, 5]. Thus, secondary prevention aimed to decrease mortality and morbidity in survivors after acute MI is of great, and increasing, significance around the world.

Platelets play a key role in the development of thrombotic and ischemic diseases [6]. Antiplatelet therapy is a major strategy for treating and preventing MI. Anti-platelet drugs have been shown to have definite and substantial net benefits for people who have occlusive vascular disease [7]. Aspirin is a safety and most cost-effective one of the anti-platelet drugs [8]. Current guidelines recommend low-dose aspirin (75–150 mg daily) for the secondary prevention of MI in many countries. However, there are several limitations related to this drug. Long-term therapy with aspirin is associated with an increase in the incidence of symptomatic peptic ulcer, duodenal ulcers, and gastrointestinal and intracranial haemorrhage, even when used at low doses or in buffered or enteric-coated formulations [9, 10]. In a population-based cohort with 4.1 million citizens in Italy, aspirin increased the risk of major gastrointestinal or cerebral bleeding episodes; patients with diabetes had a high rate of bleeding [11]. Furthermore, aspirin resistance has become a notable problem. Several studies have found that about 1 in 4 individuals may express biochemically defined aspirin resistance [12–14]. Patients who are resistant to aspirin are at greater risks of recurrent serious vascular events than those who are sensitive to aspirin [13, 14].

Current anti-platelet therapies are generally based on a specific signaling pathway in platelet activation, that is, single agent acting on single target [6]. Hence, the limitations associated with aspirin also exist for other anti-platelet agents, such as clopidogrel [6]. Agents with multipleingredients acting on multiple targets may be more effective and less harmful [15].

**Figure 1:**
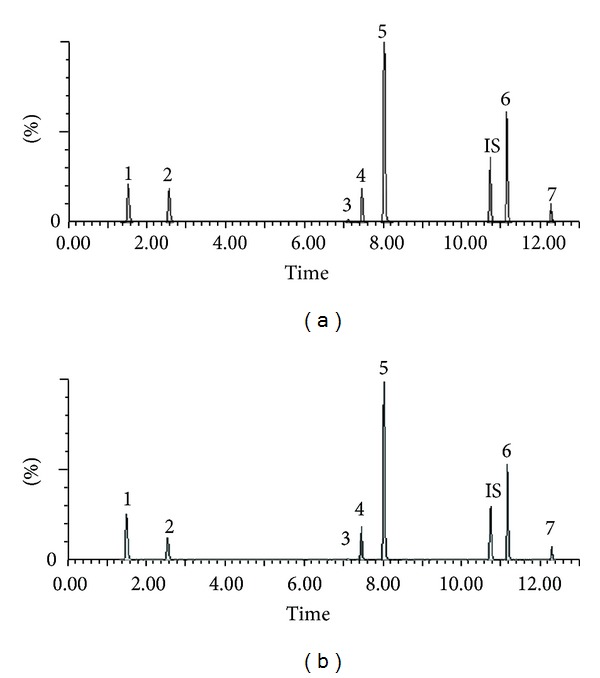
The typical chromatograms of mixed standard solution (a) and sample solution of “Qi-Shen-Yi-Qi” Dripping Pills (b). Numbers 1–7 represent danshensu, protocatechuic-aldehyde, salvianolic acid B, notoginsenoside R1, ginsenoside Rg1, ginsenoside Rb1, and astragaloside IV, respectively. IS represents digoxin. (Redrew for publication).

In Traditional Chinese Medicine (TCM), the key pathogenesis of MI is mainly “Qixu” (vital energy deficiency) and “Xueyu” (blood stagnation), that is, degradation of body function and thrombosis. Qi-Shen-Yi-Qi Dripping Pills (QSYQ), a Chinese patent medicine for adding “qi” and resolving stasis, was approved for clinical use for coronary heart disease and MI rehabilitation by the State Food and Drug Administration of China in 2003. QSYQ is made of extractions from Danshen (*Radix Salviae Miltiorrhizae*), Sanqi (*radix notoginseng*), Jiangxiang (*Lignum Dalbergiae Odoriferae*), and Huangqi (*radix astragali*). The quality control of QSYQ is good. Herbal materials were cultivated according to the Good Agriculture Practices, and manufacturing processes strictly followed the standard of Good Manufacturing Practices. UPLC-MS/MS was used to analyze seven quality control markers of QSYQ (Figure 1). There was good consistency of the active markers among different batches [16]. Pharmacological studies revealed that constituents of QSYQ could inhibit the platelet aggregation and the overrelease of *β*-TG [17]. Clinical studies have suggested that QSYQ had similar effect to aspirin in inhibiting platelet aggregation [18]. Currently, QSYQ is widely used for the secondary prevention of MI in China. However, there is insufficient evidence to know whether QSYQ can be used as an alternative to aspirin. This multicenter randomized clinical trial aimed to test whether the regular administration of QSYQ would result in a significant reduction in total serious vascular events in patients who had experienced at least one documented MI.

## 2. Methods

### 2.1. Trial Design

The clinical trial is a multi-center, randomised, double-blind, parallel controlled study. Approval was obtained from the State Administration of Traditional Chinese Medicine of China in 2004. The study protocol was reviewed and approved by the Ethics Committee in Tianjin University of Traditional Chinese Medicine. This trial was registered in the WHO Clinical Trial Registering Platform, number ChiCTR-TRC-00000002 (http://apps.who.int/trialsearch/). The study protocol was summarized here.

### 2.2. Participates

Patients with a definite diagnosis of ST-elevation or non-ST-elevation MI [19] were potentially eligible for this study if they met the following criteria: (1) the last documented MI was 4 weeks to 24 months earlier; (2) Traditional Chinese Medicine symptoms were “*Qixu-Xueyu*” (vital energy deficiency combining with blood stagnation); (3) age from 18 years to 75 years (the maximum age was adjusted from 65 years to 75 years in July 2006 because of inadequate recruitment). 

Patients were required to be free of other life-threatening diseases or problems which might have limited the ability to obtain long-time followup and to be free of any condition which would mean that regular use of the trial drugs was contraindicated. Patients with either of the following conditions were excluded: (1) a history of percutaneous coronary intervention (PCI) or coronary artery bypass graft surgery (CABG); (2) pregnant women or those who were breast feeding; (3) contraindication to aspirin (e.g., asthma, active phase peptic ulcer, and hemorrhagic disease); (4) heart function of grade IV (NYHA grade); (5) uncontrolled systemic hypertension (contractive pressure ≥180 mmHg or diastolic pressure ≥110 mmHg); (6) uncontrolled serious cardiac arrhythmias (e.g., atrial fibrillation and supraventricular tachycardia); (7) serious primary disease of liver, kidney, and hemopoietic system, or psychosis, or malignant tumor; (8) allergic history to study drugs; (9) participation in other clinical trials during the previous three months.

After a comprehensive medical evaluation, patients were given a full explanation of the study by investigators. Each patient was asked for their written informed consent before joining the study. Patients were registered if they voluntarily signed the informed consent. There was no economic compensation for participating patients. Recruitment took place in 88 hospitals throughout the east, west, south, north, and center of China. 

### 2.3. Intervention

Patients were randomly assigned to two groups (ratio 1 : 1): QSYQ group and aspirin group. In the QSYQ group, patients took 0.5 g (one package) QSYQ for three times per day and 100 mg (in four tablets) simulated enteric-coated aspirin once a day. Patients in the aspirin group took 0.5 g (one package) simulated QSYQ three times per day and 100 mg (in four tablets) enteric-coated aspirin daily. 

Patients were prohibited from taking other anti-platelet drugs or “*Yiqi-Huoxue*” Chinese medicines during the treatment period. Concomitant medications, such as antihypertensive (e.g., *β*-blockers and ACE inhibitors), hypoglycemic agents, and lipid-lowering drugs, could be prescribed at the discretion of the attending physicians and had to be recorded in detail (including drug name, beginning-stopping time, dosage, and purpose).

The treatment period for the trial drugs was 12 months. After this time (or if the trial drugs were stopped for some other reason), patients could be prescribed treatments by their physicians without any limitation. 

### 2.4. Outcome Measures

The primary endpoint was a composite of cardiovascular death, nonfatal reinfarction (documented by ECG and enzyme changes), and nonfatal stroke (diagnosed by CT or MRI). The secondary outcomes were the events of serious cardiac arrhythmias, heart failure, cardiac shock, revascularization (PCI or CABG), pulmonary embolism, and deep vein thrombosis. 

All reported adverse events during the trial were recorded to allow an assessment of the safety of the treatments. Intracranial bleedings and gastrointestinal complications that have been associated with aspirin were monitored carefully. Haemorrhagic stroke was also considered as an adverse event, although it was included in the composite primary outcome. 

### 2.5. Followup

After a first visit for collecting baseline data after randomization, enrolled patients, their dependents, or both were asked to visit clinical centers monthly. If no primary endpoints occurred, there were 12 visits during the treatment period and a final visit (6 months after the termination of the trial drugs). When a patient had one of the primary endpoints, the case was considered completed and there was no further follow-up visit. At each visit, investigators were required to retrieve and provide trial drugs; complete case report forms recording the patient's condition, endpoints, adverse events, and concomitant drugs; and remind patients not to take prohibited drugs. If a patient did not visit the clinical center at a defined time, the investigators contacted the patient or their dependents to find the reasons.

### 2.6. Quality Control Procedures

The trial was designed, executed, and analyzed by a steering committee, a clinical monitoring center, an endpoints committee, a drug management center, a data management center, and a biostatistics center. Investigators in each clinical center were trained before study beginning.

Concealment of the random allocation was achieved by using a Clinical Research Interactive Voice Respond System (CRIVRS). Investigators connected to the CRIVRS by telephone when a patient was ready to be randomised and the CRIVRS then provided a subject number, randomisation code, and drug number to the investigator by voice, email, and SMS. Because different dosage forms of two study drugs, a double-dummy design was used with placebos to blind patients and their healthcare providers. Placebos for QSYQ and aspirin were developed which had the same appearance, color, and taste as the relevant drug. One statistician who generated the blinding code was aware of the drug allocation, but patients, investigators, and other practitioners in this trial were all blinded during the whole study period.

### 2.7. Statistical Analyses

Due to a paucity of clinical data of QSYQ for the prevention of MI, the sample size for this study was estimated on the basis of practical considerations. The incident of recurred serious cardiovascular and cerebrovascular events was about 6.5% in patients who suffered from MI and used aspirin for secondary prevention [8]. It was supposed that QSYQ might have equal effectiveness compared with aspirin. The margin of equivalence (Δ) was 2.5% for primary endpoint. The sample size of 3600 subjects was calculated to be sufficient to establish equivalence (two sides *α* = 0.05 and 80% power; less than 20% loss to followup). 

Statistical analyses were conducted with SAS 9.1.3. The trial database was blind reviewed before the data were locked and unblinded. Data analyses were mainly based on the intention-to-treat (ITT) principle: with all randomized patients and all endpoints being analysed in accordance with the patients' allocated treatment group. A per-protocol (PP) analysis was performed to test the robust of the trial's results.

The baseline characteristics of the two treatment groups were compared by the *t-*test (for continuous data) and the chi-square test (for categorical data). Cox proportional-hazards regression model was used to estimate the hazard ratio (HR) and 95% confidence interval (CI) for the primary endpoint. Cumulative primary endpoints curves were constructed by Kaplan-Meier methods and differences between the curves were tested using the log-rank method. A *P* < 0.05 level (two sided) was adopted as the test for statistical significance. No subgroup analysis was prescheduled. Secondary outcome events and adverse events were analysed, and the intergroup comparisons were analyzed by using chi-square test. 

## 3. Results

**Figure 2:**
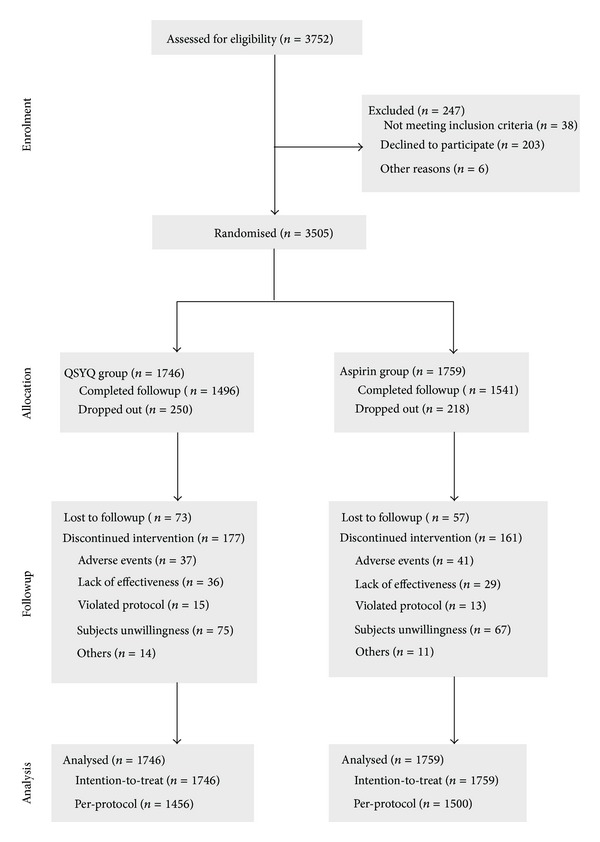
Flow diagram of participants through each stage of the trial.

A total of 3505 patients from 88 clinical centers (hospitals) throughout China were recruited and randomly assigned. There were 1746 patients in the QSYQ group and 1759 in the aspirin group (Figure 2, according to CONSORT 2010). All randomised patients were included in ITT analyses and the 2956 patients who complied with the trial protocol were included in the PP analyses.

### 3.1. Patients' Characteristics

Clinical characteristics of the included patients were shown in Table 1. In general, these baseline characteristics were similar in the two groups. The mean age of included patients was 58 years. Most of the patients were more than 12 months since their latest MI (3287 patients, 93.8%). Most of the patients had an ST-section-elevation MI, including 1109 (31.6%) anterior MIs and 1558 (44.45%) inferior MIs. About a third of patients had a body mass index (BMI) greater than 25 Kg/m^2^ and were considered to be overweight. About 70% of the participants were males. The aspirin group has a higher proportion of men than the QSYQ group which achieved statistical significance (*P* = 0.027). This imbalance for gender also led to differences in the history of smoking (*P* = 0.048) and alcohol consumption (*P* = 0.036). Therefore, gender was adjusted for sensitivity analysis, by using Cox regression analysis. There were no inter-group differences in important prognostic factors such as medical histories, concomitant medications before entry, and medications during the treatment period. 

### 3.2. Assessment of Patient Adherence

Of the included 3505 patients, 468 patients (218 in aspirin group and 250 in QSYQ group) without any recorded primary endpoint events did not complete their scheduled follow-up visits during the 12-month treatment period (Figure 2). Fifteen patients in the QSYQ group and 10 patients in the aspirin group used anti-platelet drugs for more than one week in treatment period. The figures for prohibited Chinese herbal medicines were 33 in the QSYQ group and 28 in the aspirin group. These patients who withdrew during the treatment period or who took prohibited drugs were excluded from the PP analyses. Pill counting was used to assess patients' adherence to study prescriptions and revealed an average adherence of 95% (95% of the trial drugs were taken). This suggested that patients' adherence to the trial protocol was good.

### 3.3. Primary Outcomes

Table 2 presents the primary outcome (composite endpoints of cardiovascular death, non-fatal MI, and non-fatal stroke) at the two scheduled evaluation points: after completion of 12 months of treatment and at the 6 months followup following the end of study treatments. There are a total of 104 composite endpoints after 12-month treatment, including 51 cardiovascular deaths, 37 non-fatal MIs, and 16 non-fatal strokes. When the 6-month posttreatment followup was completed, the composite endpoints increased to 131 cases, including 61 cardiovascular deaths, 52 non-fatal MIs, and 18 strokes. The 12-month and 18-month incidences of the primary outcome were 2.98% and 3.67% in the QSYQ group, respectively. The figures were 2.96% and 3.81% in the aspirin group. The differences between the two treatment groups were not statistically significant. Although minor changes to the results occurred after adjustment for gender, these changes did not make any meaningful difference to the results. 

**Figure 3:**
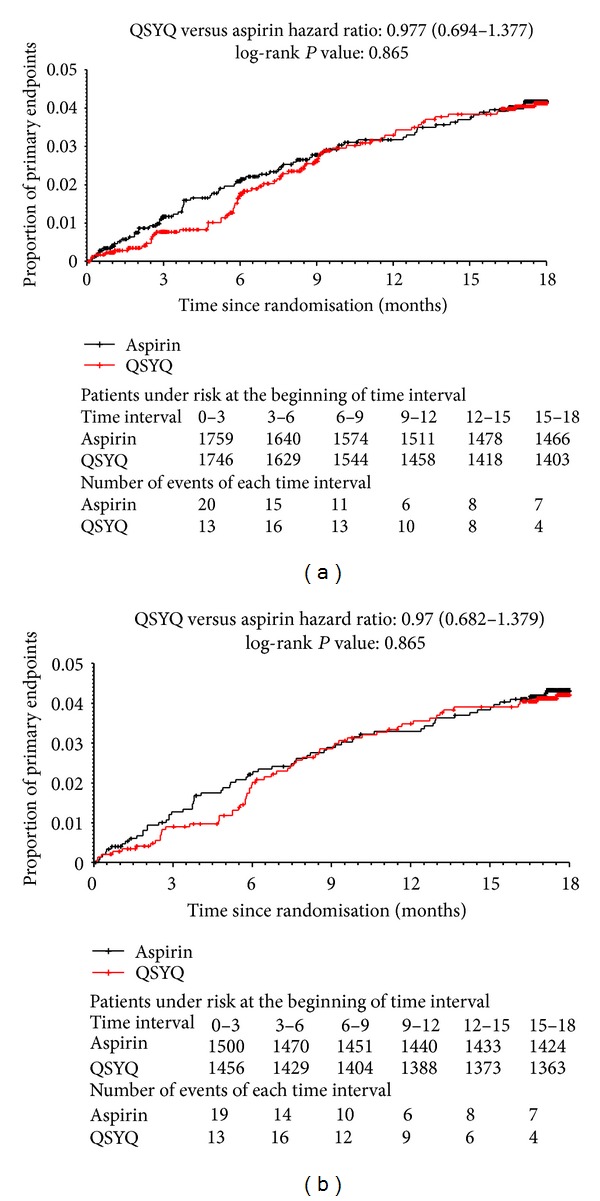
Cumulative incidence curves of the primary outcome composed of cardiovascular death, non-fatal myocardial infarction, and non-fatal stroke ((a) intention-to-treat analysis; (b) per-protocol analysis).

Cumulative analysis of the primary endpoints showed that the two curves were close during the 18-month followup (Figure 3). The hazard ratio (HR) for the primary outcome (QSYQ group versus aspirin group) was 0.977 (95% CI: 0.694, 1.377; *P* = 0.895). The PP analysis generated a similar result (HR: 0.970; 95% CI: 0.682, 1.379; *P* = 0.865).

### 3.4. Secondary Outcomes

Secondary outcome measures were shown in Table 3. During the 12-month treatment, there were a total of 42 (2.41%) secondary endpoints in the QSYQ group and 43 events (2.44%) in the aspirin group. After the 18-month followup had been completed, these incidences of secondary endpoints increased to 2.98% and 3.30%, respectively. There was no significant difference between the two groups at either of the two evaluation points.

### 3.5. Adverse Effects of Trial Drugs

Some potential adverse effects of aspirin and QSYQ were reported by patients as complaints or by the judgment of the investigator (Table 4). Nine patients in the aspirin group had a minor hemorrhage (two hemorrhagic strokes) compared to 2 patients (one hemorrhagic stroke) in the QSYQ group. There was no serious bleeding episode which required transfusion or caused death. Patients in the aspirin group had more gastric acid reflux (1.88%) than those in the QSYQ group (0.8%). This difference is statistically significant (*P* = 0.007). The incidences of stomach pain and merely allergic reactions were similar between the two groups. These data confirm that both study drugs are of good safety, and QSYQ appears to be slightly safer than aspirin.

## 4. Discussion

In this trial, QSYQ shows similar effects to aspirin for the prevention of recurrent vascular events in patient with a previous MI. The rate of composite endpoints (cardiovascular death, non-fatal MI, and non-fatal stroke) after 12-month treatment was 2.98% in QSYQ group and 2.96% in aspirin group. The incidence of serious vascular events of this trial is lower than previous studies of secondary prevention for MI [8], which may be due to several factors. 

First, this study included low-risk patients compared to previous studies: patients who had experienced PCI and CABG were excluded, and the time since latest MI was more than 12 months in most (93.8%) of the patients in this trial. Thus, the risk of recurrent vascular events might be decreased. Second, although anti-platelet agents and “Yiqi-Huoxue” Chinese herbal medicines were prohibited during the trial, other drugs including beta-blockers, ACE inhibitors, lipid-lowing drugs, and drugs for hypertension and diabetes were allowed. Third, the follow-up period is short. The three factors are likely to be the main reasons for the low incidence of serious vascular events. In the future, a study with a more broadly inclusive eligibility criteria and longer study duration should be adopted.

Modern management of myocardial infarction is built on a high quality, clinical evidence base. Anti-platelet, beta-blockers, ACE inhibitors, and statins have been recommended for clinical practice based on the results of randomized clinical trials and their systematic review and have substantially reduced mortality and morbidity associated with MI. However, most of these conventional drugs are based on specific pathways, mainly single drug acting on single target [6, 20]. This means that a patient might need to take several drugs concurrently, which leads to new problems, such as low adherence, high cost, and more adverse effects [21]. A new strategy for the management of patients following a myocardial infarction is needed. 

The concept of a “polypill” was developed about 10 years ago, with a compound pill including several recommended drugs [22, 23]. The Indian Polycap Study showed that a Polypill, composed of hydrochlorothiazide, atenolol, ramipril, simvastatin, and aspirin, had the desired effects and was safe as the individual pills [24]. Recently, a clinical trial in Sri Lanka, sponsored by the World Health Organization, has shown high acceptability of the Polypill to patients and physicians [25]. The Polypill is a new concept in western medicine but is not new in eastern medicine. QSYQ, which contains several kinds of active ingredient, is a classical polypill [26]. Over the past five years, in vivo and in vitro studies have revealed the integrated effects of QSYQ for MI, including protecting cardiac muscle cells [27], preventing cardiac ischemia-reperfusion injury via energy modulation [28], antagonizing ventricular remodeling [29], inhibiting inflammatory reaction and preventing the progress of atherosclerosis [30], and stabilizing atherosclerotic plaque by changing histological constitution [31]. A series of published studies suggested that QSYQ was a promising drug for secondary prevention of MI. However, the effects and mechanisms are not conclusive and need further studies to confirm and resolve uncertainties.

This study has some limitations. First, patients who had experienced PCI and CABG were excluded from this trial. This criterion limited the size of the sample and its representativeness. Second, biochemical measures (e.g., blood lipids) were not scheduled in this trial, in order to improve patient's adherence to followup. As a consequence, the effects of study drugs on these intermediate outcome measures were unknown. Third, our study was undertaken in China; whether the relative effects of the trial drugs would be similar in other ethnic groups is unknown. 

Our study has several strengths. This is the first multi-center trial sponsored by the national funding of China and organized by researchers of Traditional Chinese Medicine to evaluate a Chinese patent medicine for the secondary prevention of MI. Randomization and blinding were well performed by using CRIVIS. Organization, execution, and evaluation were managed by different committees which worked independently.

## 5. Conclusion

This large sample clinical trial shows that QSYQ has similar effects to aspirin in the secondary prevention of MI and has fewer adverse effects. However, the low event rates of outcome measures were insufficient to generate a confirmatory conclusion. In addition, the polypill-like effect of QSYQ should be researched step by step. Further rigorously designed studies are warranted. 

## Figures and Tables

**Table 1:** Baseline characteristics of included patients.

Proportion	QSYQ (*n* = 1746)	Aspirin (*n* = 1759)
Age at entry (years)		
Mean (SD)	58.35 (9.02)	58.28 (8.99)
≤60	910 (52.12%)	921 (52.36%)
>60	836 (47.88%)	838 (47.64%)
Gender—male*	1191 (68.21%)	1260 (71.63%)
Smoking*	773 (44.27%)	840 (47.75%)
Alcohol consumption*	505 (28.92%)	568 (32.29%)
Nationality-Han	1682 (96.33%)	1680 (95.5%)
BMI (Kg/m^2^)		
Mean (SD)	24.24 (2.82)	24.28 (2.86)
<25	1125 (64.43%)	1122 (63.79%)
≥25	621 (35.57%)	637 (36.21%)
Time since latest MI (months)		
<6	13 (0.74%)	12 (0.68%)
6–12	91 (5.21%)	102 (5.80%)
>12	1642 (94.04%)	1645 (93.52%)
Type of MI		
Non-ST-elevation MI	90 (5.15%)	69 (3.92%)
Mainly anterior MI	529 (30.30%)	580 (32.97%)
Mainly inferior MI	766 (43.87%)	792 (45.03%)
Medical history^#^		
Hyperlipidemia	683 (39.12%)	680 (38.66%)
Hypertension	753 (43.13%)	758 (43.09%)
Diabetes mellitus	219 (12.54%)	227 (12.91%)
Stroke	121 (6.93%)	106 (6.03%)
Gastritis	227 (13.00%)	212 (12.05%)
Medications before entry^†^		
Anti-platelet agents	1417 (81.16%)	1398 (79.48%)
Beta-blockers	786 (45.02%)	800 (45.48%)
ACE inhibitors	666 (38.14%)	669 (38.03%)
Lipid-lowering agents	723 (41.41%)	705 (40.08%)
Calcium blockers	238 (13.63%)	231 (13.13%)
Diuretics	107 (6.13%)	90 (5.12%)
Cardiotonic agents	40 (2.29%)	40 (2.27%)
Antiarrhythmia agents	26 (1.49%)	29 (1.65%)
Nitrates	1268 (72.62%)	1255 (71.35%)
Chinese patent medicines^§^	620 (35.51%)	636 (36.16%)

SD: standard deviation; MI: myocardial infarction; BMI: body mass index.

*The intergroup difference is statistically significant (*P* < 0.05).

^#^If a patient had more than one condition, they were counted for each of the different diseases.

^†^If a patient took more than one drug, they were counted for each of the different drugs.

^§^Chinese patent medicines were mainly used for heart disease.

**Table 2:** Composite endpoints of cardiovascular death, nonfatal myocardial infarction, and nonfatal stroke (intention-to-be-treat analyses).

Composite endpoints	QSYQ (*n* = 1746)	Aspirin (*n* = 1759)	HR (95% CI)	*P* value	HR (95% CI) Adjusted for gender	*P* value Adjusted for gender
12-month treatment completed						
Composite endpoints	52 (2.98%)	52 (2.96%)	1.02 (0.69, 1.50)	0.928	1.03 (0.70, 1.52)	0.872
Cardiovascular death	28 (1.60%)	23 (1.31%)	1.24 (0.71, 2.15)	0.450	1.26 (0.73, 2.19)	0.411
Nonfatal MI	18 (1.03%)	19 (1.08%)	0.97 (0.51, 1.84)	0.917	0.98 (0.51, 1.87)	0.952
Nonfatal stroke	6 (0.34%)	10 (0.57%)	0.61 (0.22, 1.68)	0.340	0.61 (0.22, 1.68)	0.339

6-month followup after study drugs terminated						
Composite endpoints	64 (3.67%)	67 (3.81%)	0.98 (0.69, 1.38)	0.895	0.99 (0.70, 1.40)	0.957
Cardiovascular death	31 (1.78%)	30 (1.71%)	1.05 (0.64, 1.74)	0.836	1.07 (0.65, 1.77)	0.782
Nonfatal MI	26 (1.49%)	26 (1.48%)	1.03 (0.60, 1.77)	0.925	1.04 (0.60, 1.79)	0.891
Nonfatal stroke	7 (0.40%)	11 (0.63%)	0.65 (0.25, 1.68)	0.373	0.65 (0.25, 1.68)	0.377

**Table 3:** Secondary outcome measure after 12-month treatment and 6-month followup after study drugs terminated.

Secondary endpoints	12-month treatment completed			18-month followup accomplished		
	QSYQ (*n* = 1746)	Aspirin (*n* = 1759)	HR (95% CI)	QSYQ (*n* = 1746)	Aspirin (*n* = 1759)	HR (95% CI)
Revascularization	21 (1.20%)	18 (1.02%)	1.18 (0.63, 2.20)	23 (1.32%)	26 (1.48%)	0.89 (051, 1.56)
Aggravated heart failure	20 (1.15%)	23 (1.31%)	0.88 (0.48, 1.59)	28 (1.60%)	28 (1.59%)	1.01 (0.60, 1.69)
Cardiac shock	1 (0.06%)	1 (0.06%)	1.01 (0.06, 16.09)	1 (0.06%)	2 (0.11%)	0.50 (0.05, 5.55)
Deep vein thrombosis	0 (0)	1 (0.06%)	—	0 (0)	2 (0.11%)	—

Sum up	42 (2.41%)	43 (2.44%)	0.98 (0.65, 1.50)	52 (2.98%)	58 (3.30%)	0.90 (0.62, 1.31)

**Table 4:** Adverse effects potentially associated with aspirin and Qi-Shen-Yi-Qi Dripping Pills.

Adverse effects	QSYQ group (*n* = 1746)	Aspirin group (*n* = 1759)	*P* value
Hemorrhage	2 (0.11%)	9 (0.51%)	0.06
Intracranial bleeding	1 (0.06%)	2 (0.11%)	—
Gastrointestinal bleeding	0 (0)	4 (0.23%)	—
Subcutaneous hemorrhage	1 (0.06%)	3 (0.17%)	—
Stomach pain	38 (2.18%)	37 (2.1%)	0.88
Gastric acid reflux	14 (0.80%)	33 (1.88%)	0.007
Anaphylaxis	11 (0.63%)	7 (0.40%)	0.34
